# Endoplasmic Reticulum Stress in Tuberculosis: Molecular Bases and Pathophysiological Implications in the Immunopathogenesis of the Disease

**DOI:** 10.3390/ijms26104522

**Published:** 2025-05-09

**Authors:** Jorge Sousa, Lívia Caricio Martins, Julia Moura, Amanda Pereira, Bárbara Vasconcelos, Gustavo Ferro, Pedro Vasconcelos, Juarez Quaresma

**Affiliations:** 1 Departamento de Patologia, Universidade do Estado do Pará, Belém 66050-540, Brazil; pedro.vasconcelos@uepa.br; 2 Seção de Arbovirologia e Febres Hemorrágicas, Instituto Evandro Chagas, Ananindeua 67030-000, Brazil; liviamartins@iec.gov.br; 3 Faculdade de Medicina, Universidade do Estado do Pará, Belém 66050-540, Brazil; juliamcfaria@outlook.com (J.M.); amandabeatrizp22@gmail.com (A.P.); gustavo.b.ferro@gmail.com (G.F.); 4 Hospital Universitário João de Barros Barreto, Belém 66073-000, Brazil; barbara.baldez.vasconcelos@gmail.com

**Keywords:** tuberculosis, immune system, endoplasmic reticulum stress

## Abstract

Tuberculosis (TB), caused by *Mycobacterium tuberculosis* (Mtb), is a severe pulmonary disease with high mortality, particularly in low-income countries. Early diagnosis and timely treatment, including both intensive and maintenance phases, are critical for controlling the disease and preventing its transmission. In Brazil, where TB incidence remains high, thousands of new cases are reported annually. Transmission occurs primarily through airborne droplets expelled by infected individuals. The immune response involves various cell types, such as lymphocytes and macrophages, which form granulomas to limit the spread of the bacillus. Upon entering the lungs, Mtb is phagocytosed by immune cells, where it evades destruction by blocking phagolysosome formation and inhibiting phagosome acidification. In response, the immune system forms granulomas that contain the infection, although these can become reactivated if immune function deteriorates. Mtb also interferes with host cellular organelles, particularly the endoplasmic reticulum (ER) and mitochondria, inducing cellular stress and apoptosis, which aids in its survival. Key Mtb-secreted proteins, such as BAG2 and CdhM, modulate autophagy and apoptosis pathways, influencing pathogen survival within immune cells. A deeper understanding of these molecular mechanisms, particularly the role of ER stress and its impact on immune responses, is essential for developing novel therapeutic strategies for TB prevention and treatment.

## 1. Introduction

TB is an infectious disease caused by the aerobic bacillus Mtb, which primarily affects the lungs but can involve several other organs [1,2]. Due to its ease of transmission, it is crucial to recognize the clinical signs indicative of the disease, including persistent cough with hemoptysis, low fever, night sweats, anorexia, pallor, significant weight loss, fatigue, and general weakness. Early diagnosis—confirmed through sputum smear microscopy, culture, and molecular tests—is essential for timely treatment initiation and breaking the transmission cycle [3,4]. The standard therapeutic regimen in primary care consists of two phases: the intensive phase, which aims to rapidly reduce the bacillary load and eliminate drug-resistant bacilli, and the maintenance phase, designed to reduce the risk of relapse by targeting latent or persistent bacilli [5].

TB remains the second leading infectious disease with the highest mortality rate worldwide, causing approximately 1.3 million deaths annually. An estimated 10 million new cases are reported globally each year [6]. In 2020, 9.9 million people were diagnosed with TB, and over 1.5 million died from the disease—7.5% higher than in 2019, the year the COVID-19 pandemic began [7,8]. This increase in mortality is largely attributed to the diversion of human and financial resources from TB control efforts to managing the COVID-19 health crisis [7].

It is important to note that the occurrence of TB is not solely influenced by biological factors but is also closely linked to social determinants of health. Factors such as poverty, overcrowding, and poor nutrition increase the likelihood of infection [9,10,11]. Since many underdeveloped countries face socioeconomic and environmental conditions that facilitate the proliferation of Mtb, they tend to have higher incidence rates of TB [10]. Therefore, alongside improvements in diagnostic and treatment coverage, addressing social determinants in vulnerable regions is crucial to reducing TB incidence [12,13].

In Brazil, a country with a high burden of TB, 609,837 TB cases were reported from 2012 to 2018, averaging 87,119 cases per year [8,14]. In 2019, 73,864 new cases were registered, with considerable regional variability [15]. In 2020, 67,200 new cases and 4500 deaths were recorded, placing Brazil among the top 30 countries with the highest TB burden, and ranking as the country with the most absolute cases in the Americas [8,16].

TB is highly contagious, as Mtb can remain airborne for hours, suspended in droplets expelled by an infected individual through coughing, sneezing, or even talking [1]. Following exposure to the bacillus, various factors can influence the disease progression, including coexisting conditions, environmental factors (such as ventilation and lighting), the virulence of the infecting strain, and the immunogenetics of the host. The host’s immune response to Mtb is critical in determining the course of the infection [2,17].

The body’s first line of defense against Mtb involves lymphoid tissues in the respiratory mucosa, which produce antimicrobial substances and serve as a physical barrier to limit pathogen entry. Alveolar macrophages play a key role in eliminating the bacillus through recognition, phagocytosis, and the release of inflammatory cytokines. If this initial defense is insufficient, a secondary response is initiated: peptides from the proteolysis of Mtb, present in apoptotic vesicles of macrophages, bind to the MHC class II molecule. This complex, along with co-stimulatory signals from professional antigen-presenting cells (APCs), induces clonal expansion, attracting additional immune cells, such as monocytes and lymphocytes, to the infection site to form a granuloma [18,19]. Granuloma stability is maintained by pro-inflammatory cytokines and chemokines, particularly TNF-α, which promotes leukocyte migration to the infection site [20,21]. When associated with lymph nodes, this granuloma forms Ghon’s complex, which, when visible on radiography, indicates containment of bacillary expansion and lesion development [4,22].

Regarding humoral immunity, although the exact role of B lymphocytes in producing anti-Mtb antibodies remains unclear, studies suggest their involvement in modulating and enhancing host immune responses [19]. Some studies have noted increased numbers of B lymphocytes in TB patients compared to healthy controls [2,23].

In homeostasis, the ER is crucial for protein and lipid synthesis, as well as for maintaining calcium balance within cells [24]. However, pathogen infections—whether viral, fungal, parasitic, or bacterial—can disrupt ER function, leading to the accumulation of unfolded or misfolded proteins, a condition known as ER stress [25,26]. Upon invading the host, Mtb not only interferes with ER function but also affects other organelles, such as mitochondria [27,28,29]. Recent studies have identified a novel Mtb effector protein, CdhM, which induces ER stress and macrophage apoptosis, contributing to bacillus proliferation [30,31].

## 2. General Aspects

The homeostasis of an organism depends on the function of various individual structures, or organs, and their interrelationships [32]. Similarly, the functioning of cells depends on organelles, which work to maintain the proper activity of the entire cell, one of which is the ER [33,34]. This organelle performs critical cellular functions and is classified into two types: the smooth ER, primarily involved in lipid synthesis and calcium storage, and the rough ER, which is distinguished by the presence of ribosomes, responsible for protein production and folding [35,36]. The process of protein folding requires an optimal oxidative environment, where chaperones assist in forming the specific three-dimensional structure necessary for the proper function of the protein [37,38]. Additionally, chaperones also contribute to the quality control of protein synthesis by identifying misfolded proteins and activating proteasomes to initiate their degradation [39,40].

When the folding capacity of the ER is overwhelmed, ER stress occurs, which can be triggered by the accumulation of misfolded or unfolded proteins, abnormalities in calcium storage, environmental toxins, or the presence of viral proteins [41,42]. To maintain cellular homeostasis, the ER employs a “quality control” mechanism called the Unfolded Protein Response (UPR) [43,44].

**Figure 1:**
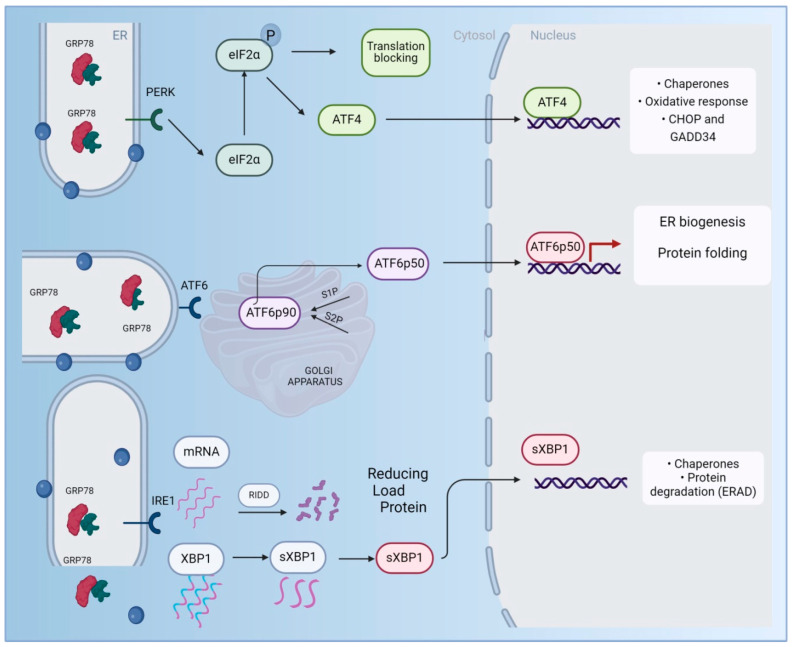
Activation mechanism of endoplasmic reticulum stress. The accumulation of misfolded proteins, which bind to GPR7, triggers the activation of three control pathways: PERK, IRE1, and ATF6. IRE1, in addition to cleaving a selected group of mRNAs to drive their manipulation through a process known as regulated IRE1-dependent decay (RIDD) to reduce the total protein-folding load in the ER, also splices XBP1, now referred to as sXBP1, which is then translocated to the nucleus, where it controls the transcription of chaperones and genes involved in protein processing. Activated PERK phosphorylates eIF2α, leading to global translation attenuation but selectively increasing the translation of the transcription factor ATF4, which translocates to the nucleus and induces the transcription of genes involved in restoring ER homeostasis. ATF6, in its ATF6p90 form, translocates to the Golgi complex, where it undergoes sequential cleavage by site 1 protease (S1P) and site 2 protease (S2P), resulting in its transcriptionally active form (ATF6p50), which initiates the transcription of genes involved in the UPR, targeting protein quality control and ER biogenesis. The UPR ultimately aims to restore ER function by blocking the synthesis of new proteins, increasing the folding capacity of accumulated proteins, and facilitating the manipulation of protein aggregates. As both the ER and mitochondria are affected by this stress, it is important to consider the potential impact on energy-dependent functions. The phosphorylation of eIF2α and the general reduction in protein synthesis may lead to ATP depletion, as cellular energy is diverted toward the stress response. This ATP depletion could affect critical cellular processes, especially those involving mitochondria. Additionally, the accumulation of misfolded membrane-bound proteins may result in histopathological changes, potentially disrupting cellular structures, including those of the ER itself. The visualization of these misfolded proteins through antibody-based staining techniques could help in assessing the extent of this damage.

The UPR consists of three main signaling pathways (Figure 1), each activated by a sensor protein that detects misfolded or unfolded proteins: ATF6, IRE1α, and PERK [45,46]. These proteins remain inactive when bound to the 78 kDa glucose-regulated protein (GRP78), a major chaperone. However, when misfolded or unfolded proteins accumulate in the ER, they displace GRP78, which then activates the sensor proteins, signaling a state of non-homeostasis [47,48]. Once activated, these pathways inhibit protein translation, increase the expression of folding chaperones, and, if proper folding is not achieved, activate proteins involved in proteasomal degradation (ERAD) [49,50]. It is important to note that in the case of chronic stress, this mechanism often fails, potentially leading to the activation of the apoptotic cascade and cell death, which is associated with the onset of pathologies such as Alzheimer’s disease [51,52].

Additionally, studies have shown that ER stress influences immune cell differentiation and activation, as well as cytokine expression, establishing a direct relationship between the proper functioning of the ER and the immune system [53,54]. The UPR also acts on immune cells, such as CD4+ T cells, where the expression of the Grp94 gene is essential for effective cell activation [55]. Studies indicate that ER stress, associated with T cell receptor (TCR) binding, increases the expression of this gene and, consequently, enhances the immune response [56,57]. Furthermore, ER chaperones, activated by this gene, play a dual role in protein folding and immune regulation [53].

The UPR also influences the differentiation of CD4+ T cells into various subtypes. Changes in the microenvironment that induce ER stress can interfere with the differentiation of naïve CD4+ T cells into their respective lineages [58,59]. Moreover, in CD8+ T cells, the UPR is triggered by pathogen presence, leading to the secretion of cytotoxic molecules and cytokines to eliminate infected cells [60]. Studies suggest that for efficient differentiation into effector T cells, the IRE1-XBP1 pathway—one of the ER stress pathways—must be activated during infection [61,62].

In B cells, the UPR is activated during differentiation, with XBP1s, a transcription factor resulting from the IRE1α pathway, playing a key role in proper protein folding and the activation of ERAD, which is important for antibody production by plasma cells. B cells also activate the IRE1-XBP1 pathway to produce large amounts of IgM [63,64]. Similarly, in macrophages, the UPR manifests similarly to B cells, where XBP1s acts as a positive regulator, amplifying responses from toll-like receptors (TLRs) on these phagocytic cells [65,66].

## 3. Mechanisms of Endoplasmic Reticulum Stress

The ER consists primarily of a network of cisternae, forming a large membranous structure composed of elongated tubules and flattened discs, occupying a significant portion of the cytoplasm. This organelle is responsible for several crucial functions: protein synthesis, calcium storage and regulation, lipid synthesis and storage, and glucose metabolism [39,67,68]. Certain conditions can disrupt ER homeostasis, a phenomenon known as “Endoplasmic Reticulum Stress” [69]. In response to this stress, the organism activates a mechanism called the UPR, which aims to restore ER homeostasis. The effectiveness of the UPR depends on the nature of the stressor, its intensity, and its duration [69,70].

One example of a condition that disrupts ER homeostasis is microenvironmental stress [71,72]. In tumors, for instance, rapidly proliferating cells lead to nutrient and oxygen depletion, creating a local microenvironmental stress. This stress results in hypoxia, acidosis, and starvation, factors that compromise ER function [68,73,74].

Additionally, exposure to recently identified ER stressors, such as tunicamycin or 2-deoxyglucose, can induce stress in the ER through different pathways [75,76]. For example, thapsigargin and cyclopiazonic acid reduce calcium concentrations in the ER, impairing its protein folding capacity [77,78].

On the other hand, exposure to agents that promote ER homeostasis can help alleviate ER stress. One such agent is 4-phenylbutyric acid (4-PBA), commonly used to reduce the accumulation of misfolded proteins in the ER. Tauroursodeoxycholic acid (TUDCA), an endogenous bile acid, is also known to act on islets to mitigate stress [79,80,81].

Furthermore, maintaining body temperature within the normal range—between 36 °C and 37 °C—is critical for overall homeostasis. Temperature deviations can lead to protein denaturation or aggregation, impairing their function [82,83].

Reactive oxygen species (ROS) synthesis and release also play a role in ER stress [84]. ROS, including free radicals, are generated during oxidative folding regulated by the UPR and in mitochondria. When ROS levels exceed the body’s natural antioxidant defenses, they can induce ER stress and promote lipid peroxidation, a process that results in the degradation of lipids in cellular membranes. This lipid peroxidation can further compromise membrane integrity, leading to increased cell membrane permeability and potential cell death [85,86]. Moreover, certain pathologies, such as tuberculosis and leprosy, cause ER stress in myeloid-derived suppressor cells (MDSCs), which suppress both innate and adaptive immunity in the host [87,88]. The misfolding of proteins during ER stress, combined with the disruption of lipid membranes due to peroxidation, poses a dual threat to the stability of the cellular environment, exacerbating the damage and contributing to the pathogenesis of these diseases.

## 4. Pathophysiology of Tb, Defense Mechanisms, and Relationship with Reticulum Stress

Mtb is the primary causative agent of human tuberculosis, a disease with significant morbidity and mortality worldwide [89,90]. Tuberculosis can manifest as primary or active disease following initial contact with the pathogen, or as reactivation disease after a period of latency, most commonly affecting immunosuppressed individuals [91,92]. The infection process involves several stages, including inhalation of the bacteria, which reach the alveolar sacs, phagocytosis by macrophages, dendritic cells, and neutrophils, blockage of phagolysosome formation to prevent bacterial death, immune responses by T lymphocytes (such as Th1), granuloma formation as an attempt to control the spread of infection, and the clinical manifestations and transmission through airborne droplets [93,94].

**Figure 2:**
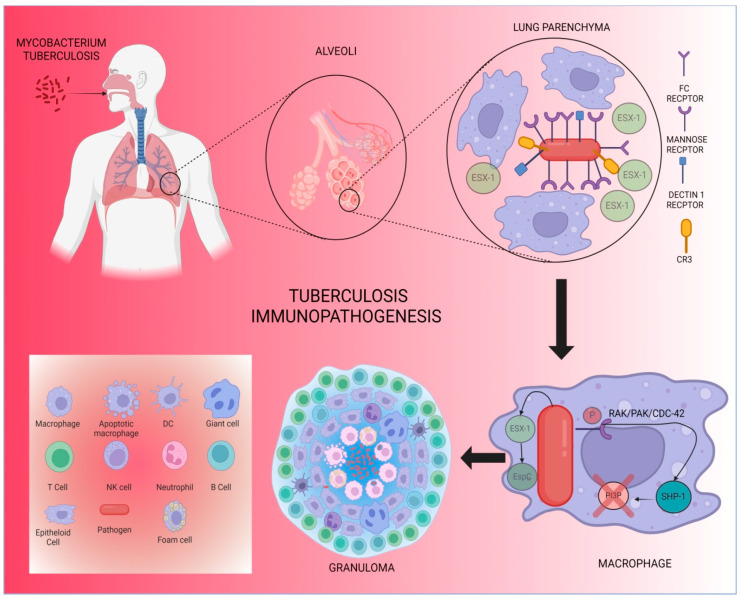
Tuberculosis immunopathogenesis. Infection occurs when Mtb enters the lung through the respiratory tract and then reaches the alveolar space, where it faces resident macrophages, which are recruited and identified by receptors (FC; mannose; dectin 1; CR3) and by the release of the ESX molecule -1, which has the EspC protein as a substrate, which increased its options within macrophages. The phosphorylated mannose receptor activates via rak/pak/cdc-4, which recruits protein tyrosine phosphatase 1 (SHP-1), which initiates the production of phosphatidylinosital 3-phosphate (P13P) in order to facilitate its specification in macrophages. The granuloma is a very characteristic histologic finding of tuberculosis, consisting of a central core composed of infected and uninfected macrophages that may be in different stages of maturation, added to neutrophils, dendritic cells, and fibroblasts, surrounded by layers of T and B cells. These macrophages can unite and form gigantocytes after transforming into epithelioid cells.

Infection by *M. tuberculosis* triggers a pro-inflammatory response in the host, primarily through the recruitment of macrophages that phagocytize the bacteria. This process is mediated by various receptors, as the surface of *M. tuberculosis* contains multiple ligands that either work together or individually to facilitate phagocytosis by specific cells [95,96]. Among these receptors, the mannose receptor is the most abundant. It activates the RAK/PAK/Cdc-42 pathway (Figure 2), which plays a role in the persistence of the bacteria within macrophages. Other receptors involved in phagocytosis include the Fc receptor, dectin-1, and complement receptors such as CR3 [93,97]. Additionally, the ability of *M. tuberculosis* to persist in the intracellular environment depends on its ability to block phagolysosome formation, thus avoiding acidification and degradation [98,99]. This process is mediated by the recruitment of Grb2 via the phosphorylated mannose receptor, which activates the RAK/PAK/Cdc-42 pathway. This, in turn, recruits protein tyrosine phosphatase 1 (SHP-1), which inhibits the production of phosphatidylinositol 3-phosphate (PI3P), a crucial regulatory lipid for phagosome–lysosome fusion, thereby facilitating the growth of *M. tuberculosis* within macrophages [100,101].

To counteract the immune evasion of *M. tuberculosis*, the host immune system employs strategies such as macrophage autophagy, which is mediated by IFN-γ produced by CD4 T cells [102,103]. This is one of the reasons why individuals co-infected with HIV are more susceptible to tuberculosis [104,105]. IFN-γ induces the production of LRG-47, a p47 GTPase that is active in phagosomal maturation and in inducing macrophage autophagy [106,107].

Granuloma formation is a hallmark histological feature of tuberculosis, consisting of a central core of infected and uninfected macrophages in various stages of maturation, along with neutrophils, dendritic cells, and fibroblasts, all surrounded by layers of T and B cells (Table 1) [108,109]. These macrophages can fuse to form multinucleated giant cells, transitioning into epithelioid cells [110,111]. The hypoxic environment within the granuloma induces necrotic cell death, leading to the formation of a caseous center [112,113]. Granulomas serve as a mechanism to limit the long-term spread of the infection, but they are also susceptible to reactivation in cases of immunosuppression. *M. tuberculosis* can further promote its proliferation by producing the ESX-1 protein, which recruits macrophages to facilitate its growth [114,115,116].

In addition to ESX-1, *M. tuberculosis* produces the substrate EspC, which induces apoptosis of macrophages through ER stress [117,118]. Studies have shown that overexpression of EspC increases the viability of mycobacteria within macrophages, as this substrate induces the unfolding of proteins that accumulate in the ER, leading to organelle stress [119,120]. EspC upregulates the transcription of key ER stress markers such as CHOP, Bip, eIF2α, and PERK [121,122]. Subsequently, apoptosis is triggered through the activation of caspase-12, caspase-9, and caspase-3 [123,124].

CDP-diglyceride hydrolase (CdhM) is another *M. tuberculosis* effect or protein found in granulomas involved in ER stress by activating the UPR [125,126]. ER stress markers, such as unconventional splicing of XBP-1 mRNA via the IRE1 pathway and increased phosphorylation of eIF2α via the PERK pathway, have been detected in macrophages from caseous granulomas, suggesting that ER stress-induced apoptosis in tuberculosis occurs via at least these two UPR pathways (Table 1) [125,127]. Consequently, this mechanism promotes the increased dissemination of *M. tuberculosis* within caseous granulomas [128,129].

## 5. Immune Evasion Strategies Related to Reticulum Stress

To survive in a hostile cellular environment, pathogenic bacteria employ various immune evasion strategies, such as inhibition of apoptosis and suppression of autophagy [130,131]. Several studies have shown that Mtb can inhibit apoptosis in infected macrophages and instead induce necrosis to prevent bacterial death [132,133,134].

**Figure 3:**
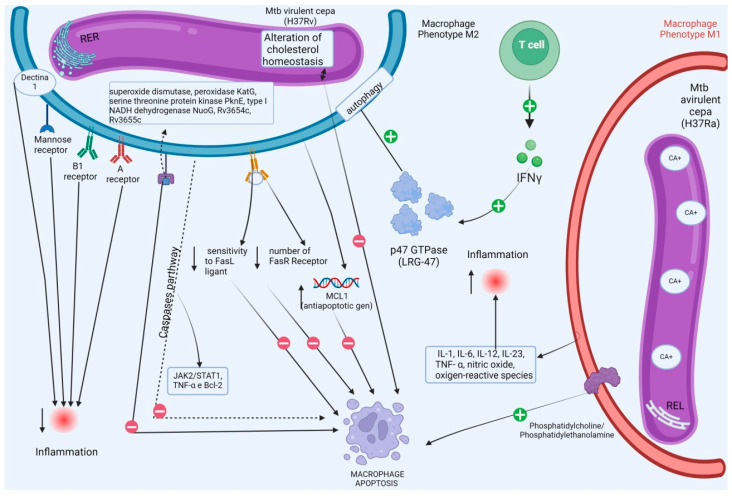
Maturation implications of response polarization (macrophages) and its correlation with reticulum stress via *M. tuberculosis*. IFN-γ, produced by CD4 T cells, induces the production of LRG-47, a p47 GTPase, active in phagosomal maturation and induction of macrophage autophagy. Macrophages infected with the virulent strain H37Rv (phenotype M2) present predominantly rough ER, which demonstrates inhibition of the apoptosis process by inhibition of the caspase pathway (JAK2/STAT1, TNF-α, Bcl-2), alteration of cholesterol homeostasis and FasL ligand, correlating with a greatly reduced expression level of FasR receptors, in addition to positive intervention in the expression of the antiapoptotic gene MCL1. The avirulent strain H37Ra (phenotype M1), which presents a smooth ER phenotype, presents an increase in cytosolic calcium and simultaneous stimulation of phosphatidylcholine/phosphatidylethanolamine expression, which stimulates macrophage apoptosis.

From this perspective, to escape apoptosis, Mtb manipulates the ultrastructural machinery of the macrophage ER [130,135]. For example, macrophages infected with the virulent H37Rv strain exhibit a predominantly rough ER compared to macrophages infected with the avirulent H37Ra strain, which display a smooth ER phenotype. The consequences of this phenotype change result in increased cytosolic calcium levels and the simultaneous stimulation of phosphatidylcholine/phosphatidylethanolamine expression in H37Ra-infected macrophages, which facilitates apoptosis [136,137]. In macrophages infected with the H37Rv strain (Figure 3), cholesterol homeostasis is altered, inhibiting apoptosis and promoting persistent infection [138,139].

Moreover, macrophages infected with the H37Rv strain show reduced susceptibility to FasL ligand-induced apoptosis, correlating with a significantly decreased level of FasR receptor expression [140,141]. These virulent strains may interfere with TNF signaling by upregulating the expression of an anti-apoptotic gene called MCL1, which belongs to the BCL2 gene family [138,142].

The ER stress response plays a significant role in limiting Mtb survival by inducing apoptosis. Depending on the initial stimuli, macrophages may acquire a distinct phenotype during the polarization process due to calcium imbalance [143,144]. These phenotypes are classified as M1 or M2 macrophages, also known as classically or alternatively activated macrophages [145]. Classical activation, stimulated by microbial products, leads to M1 macrophages, which are characterized by high antigen presentation and production of cytokines such as IL-1, IL-6, IL-12, IL-23, TNF-α, nitric oxide, and reactive oxygen species intermediates [146]. These M1 macrophages promote an increased inflammatory response and mediate resistance against intracellular pathogens [147].

In contrast, alternative/M2 activation reduces inflammation, modulates the immune response, and regulates surface molecules such as dectin-1, the mannose receptor, and deletion receptors A and B1. M2 macrophages also produce high levels of IL-10 [147]. Macrophages infected with the attenuated H37Ra strain exhibit an M1 phenotype, while macrophages infected with the virulent H37Rv strain predominantly display an M2 phenotype [146,147]. ER stress stimulates macrophage polarization towards M1, facilitating pathogen clearance, indicating that ER stress may be an essential component of the host immune response. For Mtb to survive, it must evade these cell death processes [138,139].

Additionally, several proteins secreted by virulent Mtb strains, such as superoxide dismutase, hydrogen peroxide/peroxidase KatG, serine threonine protein kinase PknE, I-type NADH dehydrogenase NuoG, Rv3654c, and Rv3655c, have been shown to inhibit macrophage apoptosis [135]. These proteins regulate nitric oxide and pro-inflammatory cytokine production, disrupting the relationship with Toll-like receptors [136]. As a result, Mtb inhibits TNF-α-induced apoptosis and interferes with caspase pathways, including JAK2/STAT1, TNF-α, and Bcl-2, further reducing macrophage apoptosis and enhancing bacterial survival [114,131].

The coexistence of Mtb and the host is made possible by the above mechanisms. The early secretory proteins CFP-10 and ESAT6 regulate macrophage apoptosis at various stages of infection by modulating TNF-α levels [147]. Moreover, Mtb induces IL-10 release from immune cells such as macrophages, monocytes, B cells, cytotoxic T cells, and NK cells, while elevating TNFR2 release, thereby restricting TNF-α activation [140]. Furthermore, the transmembrane protein Bcl-2 plays an essential anti-apoptotic role, controlling the transport of intra- and extracellular substances, inhibiting calcium release, and/or blocking peroxide accumulation in the intracellular environment, thereby escaping apoptosis and the immune response [114].

## 6. Influence of ER on the Cell Death Process Against *M. tuberculosis*

Cytoprotective mechanisms are essential for maintaining organism homeostasis, requiring the regulation of cellular processes to preserve cellular balance [115]. Autophagy is an important cellular process in the defense against pathogens such as *M. tuberculosis* and is mediated by multifunctional proteins of the BAG family, specifically BAG2, which is responsible for selective autophagy of cytoplasmic organelles [147]. This occurs through the dissociation of the BCL2-BECN1 complex by BAG2, which phosphorylates the anti-apoptotic protein BCL2, triggering autophagy in infected cells [139].

The expression of the BAG2 protein is regulated by mycobacteria-containing cells due to their induction of ER. Following the activation of the UPR, which involves three signaling pathways—IRE1, ATF6, and PERK—there is a decrease in BAG2 expression [125]. This decrease triggers the apoptotic process, modifying the relationship between autophagy and apoptosis, ultimately leading to cell death. In this context, the IRE1 signaling pathway plays a fundamental role in apoptosis, as its decrease results in increased BAG2 expression. Activation of the IRE1 pathway, in turn, activates the transcription factor XBP1, which inhibits the production of proteins in the ER through unconventional splicing of mRNA and consequently inhibits the BAG2 promoter [140].

Another mechanism responsible for ER stress-induced apoptosis in *M. tuberculosis* infection is the effector protein CdhM [117]. This process aids in the dissemination of *M. tuberculosis* by promoting macrophage apoptosis [118]. CdhM induces increased levels of Bip and CHOP, markers of ER stress, as well as increased XBP1 splicing, a process similar to the one described above [119]. Thus, ER stress significantly influences the cell death process during *M. tuberculosis* infection, mediated by two mechanisms: the inhibition of BAG2 protein through activation of the IRE1 pathway and by the effector protein CdhM [118,119].

Apoptosis in *M. tuberculosis* infection has dual functions. It can be beneficial in the early stages of infection to control the pathogen but can become detrimental in later stages, as macrophage death may lead to the dissemination of infectious agents. Therefore, understanding these molecular mechanisms is crucial for developing effective prevention strategies and treatments [120].

## 7. Conclusions

This study sheds light on the functioning of the ER and its protective mechanisms in response to disturbances in cellular homeostasis. It highlights various factors that can induce stress in this organelle, such as microenvironmental changes, exposure to stressors, high levels of reactive oxygen species, altered body temperature, and pathologies like tuberculosis, which is the focus of this research.

Mtb employs infection strategies such as proliferation within macrophages, evading phagolysosome formation. This leads to granuloma formation as part of the body’s defense system, with IFN-gamma release serving as a mechanism to contain the spread of the bacteria. In this context, Mtb regulates markers of UPR pathways associated with ER stress through the secretion of substances like Esx-1 to evade the host’s immune system.

Moreover, immune evasion strategies are intricately linked to ER stress, with pathogenic bacteria employing various mechanisms to inhibit apoptosis and suppress autophagy. To evade apoptosis, *M. tuberculosis* manipulates the ultrastructural machinery of the macrophage ER, as demonstrated in the virulent H37Rv strain. Additionally, several proteins secreted by Mtb, including superoxide dismutase, KatG, and PknE, have the ability to inhibit macrophage apoptosis, further enhancing bacterial survival in the host.

Future research could explore the potential of targeting ER stress pathways in the treatment of Mtb infection. Modulating UPR signaling or enhancing autophagic responses could offer novel therapeutic avenues, particularly in the context of latent tuberculosis infection or chronic disease. Furthermore, understanding the role of ER stress in different stages of infection could reveal critical insights into optimizing therapeutic strategies for early, late, or latent stages of tuberculosis.

## Figures and Tables

**Table 1:** Implications of ER stress response in tuberculosis pathogenesis.

Aspects	Immunoprotective Effects of ER Stress	Immunopathogenic Effects of ER Stress	References
Immune signaling and cytokine activation	Infection by *M. tuberculosis* induces initial ER stress, activating sensors such as IRE1α, PERK, and ATF6, which promote the expression of IL-6, TNF-α, and IFN-γ via NF-kB and XBP1s, strengthening the TH1 response and M1 macrophage activation, essential for restricting bacterial replication in the early stages of infection.	The persistence of *M. tuberculosis* in the intracellular environment, by chronically deregulating the UPR pathway, tends to favor immunosuppression and induce an imbalance in Th1/Th2 responses, contributing to increased bacterial burden.	Cui et al., 2016 [107] Lim et al., 2016 [108]
Intracellular bacterial control	ESAT-6 activates the endoplasmic reticulum (ER) stress pathway, leading to the expression of key proteins such as GRP78, CHOP, and phosphorylation of eIF2α. The induction of these proteins is related to activation of the eIF2α/ATF4/CHOP pathway, resulting in increased Ca^2+^ concentration and ROS production, contributing to the microbicidal response.	One immune evasion strategy involves ER stress induction by CdhM, which may facilitate the release of *M. tuberculosis* from macrophages, promoting infection dissemination and prolonging its intracellular survival.	Xu et al., 2022 [116] Choi et al., 2010 [53]
Granuloma formation and stability	Cellular stress induced by *M. tuberculosis* infection leads to the coordinated release of chemokines (CCL2, CXCL10) and adhesion molecules (ICAM-1), favoring cell recruitment and the formation of structured granulomas that contain the infection.	*M. tuberculosis* can exploit chronic ER stress to induce the expression of CHOP/GADD153/Ire1α/ATF3 and promote uncontrolled cell death, destabilizing the granuloma, favoring its rupture, and facilitating bacillary dissemination.	Domingo-Gonzalez et al., 2016 [117] López Ramírez et al., 1994 [118] Seimon et al., 2010 [119]

## Data Availability

Not applicable.
